# The relationship of weather with daily physical activity and the time spent out of home in older adults from Germany – the ActiFE study

**DOI:** 10.1186/s11556-022-00286-0

**Published:** 2022-02-12

**Authors:** Matthias Klimek, Raphael Simon Peter, Michael Denkinger, Dhayana Dallmeier, Kilian Rapp, Dietrich Rothenbacher, Jochen Klenk, the ActiFE study group, B. Böhm, H. Geiger, A. Lukas, J. Stingl, M. Riepe, K. Rapp, K. Scharffetter-Kochanek, W. Koenig, J. M. Steinacker, A. Ludolph, G. Nagel, R. Peter

**Affiliations:** 1 Department of Clinical Gerontology, Robert-Bosch-Hospital, Auerbachstr 110, 70376 Stuttgart, Germany; 2 Institute of Epidemiology and Medical Biometry, Ulm University, Helmholtzstr 22, 89081 Ulm, Germany; 3 Institute of Geriatric Research, Ulm University, Helmholtzstr 22, 89081 Ulm, Germany; 4 Agaplesion Bethesda Hospital, Geriatric Research Unit, Zollernring 26, 89073 Ulm, Germany; 5 Geriatric Center Ulm/Alb-Donau, Ulm, Germany; 6 Department of Epidemiology, Boston University School of Public Health, 715 Albany Street, Boston, USA; 7 IB University for Health and Social Sciences, Study Centre Stuttgart, Paulinenstraße 45, 70178 Stuttgart, Germany

**Keywords:** Older people, Accelerometer, Sensor-based, Movement diary, Weather, Physical activity, Time out-of home, Generalized mixed models, Generalized additive models, Splines

## Abstract

**Background:**

There is a need for a comprehensive evaluation of the associations between varieties of weather conditions on the time spent out-of-home (TOH) and on walking duration (WD) among older adults. We aim to investigate the extent to which various weather parameters (temperature, solar radiation, sunshine duration, humidity, windspeed, and rain) determine daily WD the TOH in older adults.

**Methods:**

The ActiFE (Activity and Function in Older People in Ulm) study is a prospective study of participants aged 65 years or older who wore an accelerometer and kept a movement diary in up to three temporally separated waves from 2009 to 2018 for a duration up to seven days per wave (up to three weeks in summary). We used weather data from a weather station near the participants‘ homes. Age-adjusted and gender-stratified generalized mixed models were used to predict WD and TOH (with 95% confidence interval (CI)) within and between weather categories. Generalized additive models were computed for the single predictions at the weather quartile boundaries. Cubic splines (with 95% pointwise confidence bands (CB)) visualized the continuous course of the weather values.

**Results:**

Higher temperatures, solar radiation and more hours of sunshine, led to an increase in WD and TOH, while higher precipitation, humidities and windspeeds led to a decrease. Women had in general higher WD and TOH times than men.

**Conclusions:**

Our data suggest that weather parameters have a considerable influence on PA and TOH. Future analyses and interpretation of PA data should therefore account for weather parameters.

**Supplementary Information:**

The online version contains supplementary material available at 10.1186/s11556-022-00286-0.

## Background

Physical activity (PA) is associated with many positive health benefits including improvement of many health and functional parameters [1]. Strong evidence exists that at least moderately active subjects have a lower risk for various chronic diseases such as cardiovascular disease, diabetes, cancer, bone diseases, and others [2, 3]. In addition, PA and outdoor activity can have social and psychological benefits. Preventive effects for several pathological conditions have been documented in adolescents and seniors alike: Vitamin D deficiency, multiple sclerosis, osteoporosis and myopia [4]. Furthermore, previous studies showed that older adults with a longer walking duration (WD) or who spend more time out-of-home (TOH) have a lower mortality risk [5–7].

Potentially important determinants of PA, especially outdoor PA, are weather conditions. Several studies in older adults applying accelerometer measurements have shown that higher daily temperatures as well as longer day lengths combined with pleasant weather are associated with higher PA [8–11]. In addition, unpleasant weather decreases physical activity in rather untrained older adults, compared to very fit persons [12]. After controlling for relevant covariates, low sunshine duration, very low and extremely high daily temperatures, and light to heavy rainfall all have been related to lower PA [13].

Most studies regarding weather parameters are limited to the daily temperature, the sunlight and sometimes also the precipitation. Therefore, it is of interest to look more closely into further weather parameters like solar radiation, humidity or windspeed. Since exposure to weather conditions occurs outdoors, TOH may be an important intermediate factor between weather conditions and PA. To the best of our knowledge, there has neither been a comprehensive evaluation of the associations between varieties of weather conditions on TOH nor on WD among older adults.

The aim of the study was to investigate the extent to which various weather parameters (temperature, solar radiation, sunshine duration, humidity, windspeed, and rain) are associated with sensor-based measurements of PA (daily WD) and diary-based daily TOH in community-dwelling older adults participating in the ActiFE study (Activity and Function in Older People in Ulm).

## Methods

The ActiFE-Ulm study is a population-based longitudinal cohort study to analyze the association of accelerometer-based physical activity and its change with different health- and disability-related parameters. Between 2009 and 2010, 7624 non-institutionalized community-dwelling older adults aged 65 years or older were randomly selected from the Ulm inhabitant registry (“Einwohnermeldeamt”) and adjacent regions and contacted for participation. Exclusion criteria were inability to walk, nursing care home residents, cognitive difficulties, or difficulties in understanding the German language. A total of 1506 eligible participants (participation rate: 19.8%) gave their consent to participate in the study. Details of the measurements collected have been described previously [14].

The final study population included all subjects with activPAL measurements (from which PA emerged) and valid data in the diary-based daily TOH. Standardized assessments of participants were collected during baseline (years 2009/2010) and two follow-up waves conducted 2012/2013 and 2016/2017 containing among others medical history information on physician-diagnosed comorbidities and symptoms (e.g., stroke, heart attack and diabetes, etc.). After excluding those who declined further participation, 931 (61.8%) and 665 (44.2%) subjects attended the 3- and 7-years follow-up, respectively. We observed 359 deaths (23.8%) at the 7-years follow-up.

In order to investigate the influence of weather on the outcome variables WD and TOH, we used weather data from the closest available weather station in the recruitment region of our participants (located in Weißingen; about 15 km from the center of Ulm/Neu-Ulm). This weather data was provided from the agricultural meteorolody database of the State of Bavaria as a CSV file [15]. The exposure variables included the maximum temperature in degrees Celsius (measured by a thermometer at a height of two meters over the ground), the sunshine duration (measured by incident sunlight on a kind of glass sphere), the accumulated amount of solar radiation in kilowatts per hour on a square metre per day (measured by a pyranometer at a height of two meters over the ground), the accumulated amount of daily precipitation in millimetres per hour (measured by the amount of rain captured at a height of 50 cm over the ground), the daily average humidity in percent (measured by a hygrometer at a height of two meters over the ground), the average windspeed in metres per second (measured by a rotating anemometer at a height of two meters over the ground). For the analyses, we categorized each weather parameter values into quartiles.

**Fig. 1:**
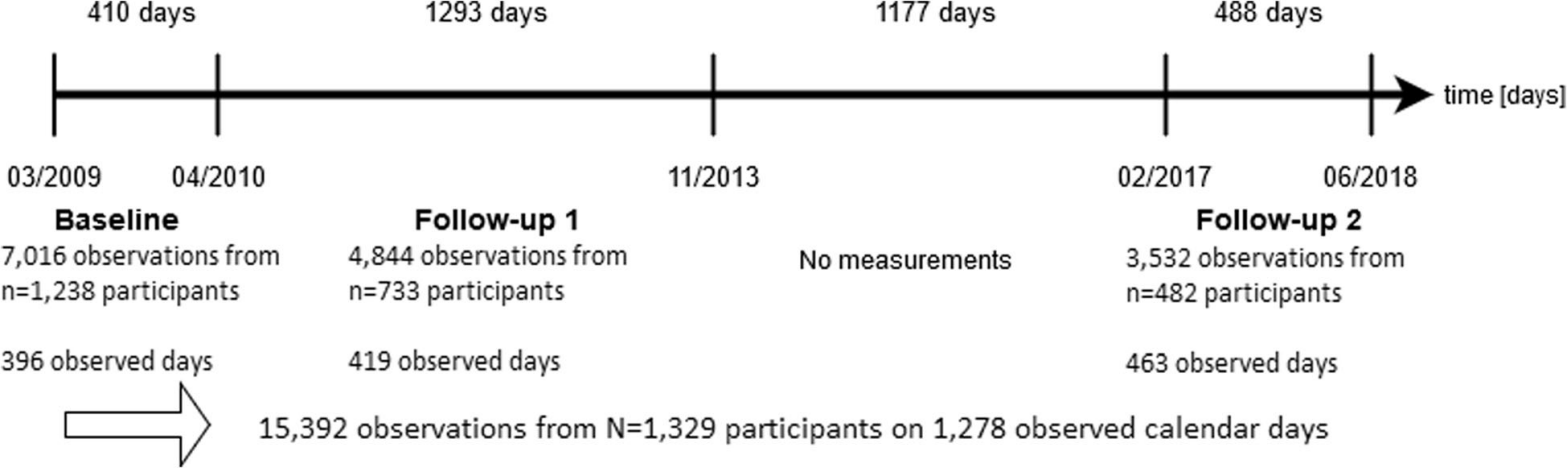
Timeline of the three waves (baseline and two follow-ups)

The activPAL sensor (PAL Technologies Ltd., Glasgow, UK) recorded physical activity 24 h a day at a sampling rate of twenty times per second and detected whether someone had been walking and for how long. The participants were advised to wear the sensor for the full measurement period of one week. Only full days with 24 h were considered for our analyses. Due to technical problems or compliance issues, not all participants achived the same number of measurement days. The minimum sensor wearing time for included participants was one day. Nevertheless, the median wearing time per wave was 6 days. From these recordings, the WD of each measurement day of each participant was calculated. Along with the sensor-based measurement, the participants completed a movement diary at what time during the day they left the house and returned. The sum of these self-reported periods of time resulted in the sum of the TOH. On days when participants were not outside, the sum of TOH was zero, which was also included in the analyses. Therefore, every single day of a participant represents an observed unit. A detailed representation of the timeline, including the number of observed days, the number of participants and the included calendar days, is shown in Fig. 1.

Generalized mixed models (GMM) were used to account for the structure of the repeated measurements. We adjusted for participants’ age at measurement day to incorporate the time difference between the measurements into the models. The association between the categorized weather values and both outcomes was calculated stratified by sex. The predicted means and their 95% confidence intervals (CI) were estimated from these models. In addition, we calculated the differences in predicted means (along with 95% CIs) between the next higher categories to compare the change in the outcomes. However, due to the different number of days observed in each weather category, the number of differences calculated was not equal.

In order to show the continuous course of the weather values, generalized additive mixed models (GAM) were calculated to present graphically restricted cubic splines with five knots at their quartile boundaries (at 0, 25, 50, 75 and 100%) with corresponding 95% pointwise confidence bands (CB). We likewise calculated the prediction for the weather parameters at their quartile boundaries to bring extreme values into closer focus. Due to the high density of rain values equal to zero, only classifying the rain values into a split at the 75% percentile was feasible. Due to a small number of observations, we truncated the upper 5% of the precipitation and windspeed values and the lower 5% of the humidity values in the graphs. All analyses were performed using R version 4.0.2.

## Results

The analysis population included 1329 participants with PA data from at least one of the three investigated time periods; women comprised 44.4% (590 participants). During all time periods we obtained data during unique 1278 calendar dates (Fig. 1) representing 15,392 observed days for the analyses. Mean age of participants across all observed days was 77.1 years [standard deviation (SD): 6.4]. On 18.6% (2862 days) of the total observed days, 75.6% (*n* = 1005) of the participants spent at least one day only at home.

As displayed in Table 1, across the entire follow-up time, participants walked an average of 100.9 min (SD: 48.7) per day. Stratified by sex, men walked an average of 99.0 min (SD: 49.0) and women walked an average of 103.4 min (SD: 48.2) per day. All participants spent an average of 197.0 min (SD: 165.1) TOH per day across all waves. In gender comparison, women had been out-of-home for 198.3 min (SD: 161.6) and men for 196.0 min (SD: 167.8). During the observation period, the average temperature was 12.6 °C (SD: 9.7), global radiation was 3331 kWh/m^2^ (SD: 2402), average duration of sunshine was 5.6 h (SD: 4.6), daily precipitation was 2.0 mm/h (SD: 4.4), humidity was 86.0% (SD: 9.2), windspeed was 1.5 m/s (SD: 1.0) per day. Most of the considered weather parameters were correlated (**Supplemental Table A.**1). The highest Spearman correlation coefficients were observed for average daily temperature, global radiation, average duration of sunshine and humidity (rho between 0.49 and 0.91). Additionally, we analyzed different mutually adjusted models including those parameters of different domains and those with a low risk of collinearity (i.e. temperature, rain, windspeed). However, the explained variance did not increase significantly (Supplemental Table A.2). This table also shows the estimators in the mutually adjusted models.

**Table 1:** Participants characteristics for the analysis population

	Analysis population ***N*** = 1,329	Women ***n*** = 590 (44.4%)	Men ***n*** = 739 (55.6%)
Included observed days (total), *n*	15,392	6655	8737
- At baseline, *n*	7016	3130	3886
- At follow-up 1, *n*	4844	2068	2776
- At follow-up 2, *n*	3532	1457	2075
Days not spent out-of-home, *n* (%)	2862 (18.6%)	1158 (17.4%)	1704 (19.5%)
- No. of participants (%)	1005 (75.6%)	432 (73.2%)	573 (77.5%)
Age (years), mean (SD)	77.1 (6.4)	77.2 (6.4)	77.0 (6.3)
- At baseline, mean (SD)	75.4 (6.5)	75.5 (6.5)	75.2 (6.5)
- At follow-up 1, mean (SD)	77.2 (6.1)	77.5 (6.2)	77.0 (6.0)
- At follow-up 2, mean (SD)	80.3 (5.1)	80.3 (5.2)	80.2 (5.0)
Lower education, *n* (%)	741 (56.5%)	334 (57.3%)	407 (55.8%)
BMI (kg/m^2^), mean (SD)	27.6 (4.1)	27.5 (4.1)	27.4 (4.1)
Hypertension, *n* (%)	659 (53.3%)	292 (52.8%)	367 (53.7%)
Myocardial infarction, *n* (%)	116 (9.4%)	48 (8.7%)	68 (9.9%)
Stroke, *n* (%)	62 (5.0%)	30 (5.4%)	32 (4.7%)
Diabetes, *n* (%)	170 (13.7%)	79 (14.3%)	91 (13.3%)
Daily walking duration (min), mean (SD)	100.9 (48.7)	103.4 (48.2)	99.0 (49.0)
Daily time out-of-home (min), mean (SD)	197.0 (165.1)	198.3 (161.6)	196.0 (167.8)
Daily maximum temperature (°C), mean (SD) [Min-Max]	12.6 (9.7) [−8.5–35.00]		
Daily solar radiation (kWh/m^2^), mean (SD) [Min-Max]	3331 (2,402) [127–9,488]		
Daily hours of sunshine, mean (SD) [Min-Max]	5.6 (4.6) [0.0–16.5]		
Daily rain (mm/h), mean (SD) [Min-Max]	2.0 (4.4) [0.0–45.5]		
Daily humidity (%), mean (SD) [Min-Max]	86.0 (9.2) [53.8–93.6]		
Daily windspeed (m/s), mean (SD) [Min-Max]	1.5 (1.0) [0.0–6.0]		

Legend: Comorbidities and symptoms at baseline (baseline population: *n* = 1238). All other results refering to the data of all three investigation periods

As shown in Tables 2 and 3, there was a continuous increase in WD and TOH for men and women across quartiles for temperature, radiation and sunshine duration. For WD the increase was similar in men and women, while, on average, men spent longer TOH in the two upper quartiles and shorter TOH in the two lower quartiles of temperature, radiation and sunlight when compared to women.

**Table 2:** **Mean predictions of WD and TOH within the weather parameter categories and mean predicted differences between the categories stratified by women**

Women Predicted mean (95% CI)	Q1	Difference Q2-Q1	Q2	Difference Q3-Q2	Q3	Difference Q4-Q3	Q4
**Temperature** (°C)	≤4.5		> 4.5- ≤ 12.9		> 12.9- ≤ 20.5		> 20.5
Daily WD (min)	95.0 (94.4; 95.7)	**6.2 (5.2; 7.1)**	101.0 (100.4; 101.7)	**4.2 (3.3; 5.0)**	105.6 (105.0; 106.2)	**6.1 (5.2; 7.0)**	111.7 (111.1; 112.3)
Daily TOH (min)	166.1 (164.5; 167.7)	**22.9 (20.7; 25.2)**	188.7 (187.1; 190.3)	**17.7 (15.6; 19.9)**	207.4 (205.8; 208.9)	**22.0 (19.8; 24.3)**	229.5 (227.9; 231.1)
**Radiation** (kWh/m^2^)	≤1170		> 1170- ≤ 2770		> 2770- ≤ 5190		> 5190
Daily WD (min)	96.6 (95.9; 97.2)	**2.7 (1.8; 3.6)**	99.2 (98.6; 99.9)	**5.1 (4.2; 5.9)**	104.5 (103.8; 105.1)	**8.4 (7.6; 9.3)**	112.9 (112.3; 113.5)
Daily TOH (min)	179.0 (177.4; 180.6)	**7.8 (5.7; 9.9)**	186.6 (185.1; 188.2)	**14.5 (12.4; 16.6)**	201.5 (200.0; 203.1)	**23.6 (21.4; 25.8)**	225.1 (223.6; 226.6)
**Sunshine** (h)	≤1.3		> 1.3- ≤ 5.0		> 5.0- ≤ 9.3		> 9.3
Daily WD (min)	95.7 (95.1; 96.4)	**4.3 (3.4; 5.2)**	100.2 (99.6; 100.8)	**4.9 (4.0; 5.8)**	104.9 (104.2; 105.5)	**7.6 (6.7; 8.5)**	112.4 (111.8; 113.1)
Daily TOH (min)	179.9 (178.3; 181.5)	**10.4 (8.3; 12.5)**	190.7 (189.2; 192.2)	**11.8 (9.7; 13.9)**	201.9 (200.4; 203.4)	**18.3 (16.2; 20.4)**	220.1 (218.6; 221.6)
**Humidity** (%)	≤80		> 80- ≤ 87		> 87- ≤ 94		> 94
Daily WD (min)	108.7 (108.1; 109.3)	**−3.3 (−4.2; −2.5)**	105.1 (104.5; 105.8)	**−4.1 (−5.0; −3.2)**	101.2 (100.5; 101.8)	**−3.4 (− 4.3; − 2.4)**	97.8 (97.1; 98.5)
Daily TOH (min)	209.8 (208.3; 211.2)	**−6.9 (−8.9; − 4.8)**	202.2 (200.7; 203.7)	**−9.2 (−11.4; −7.1)**	193.5 (192.2; 195.0)	**−7.4 (−9.6; − 5.1)**	186.1 (184.4; 187.7)
**Windspeed** (m/s)	≤0.8		> 0.8- ≤ 1.3		> 1.3- ≤ 2.0		> 2.0
Daily WD (min)	106.5 (105.9; 107.1)	**−1.1 (−2.0; −0.2)**	105.1 (104.5; 105.8)	**−1.7 (− 2.6; −0.9)**	103.4 (102.8; 104.0)	**−5.2 (−6.1; − 4.3)**	98.1 (97.4; 98.7)
Daily TOH (min)	212.5 (211.0; 214.0)	**−6.2 (−8.3–4.2)**	205.6 (204.1; 207.1)	**− 8.1 (− 10.1; − 6.1)**	197.5 (196.0; 199.1)	**−21.1 (− 23.2; − 18.9)**	176.0 (173.4; 177.6)
**Rain**^**a**^ (mm/h)	≤1.6		> 1.6				
Daily WD (min)	104.5 (104.2; 104.9)	**−4.6 (− 5.1; − 4.1)**	99.8 (99.1; 100.4)				
Daily TOH (min)	199.8 (199.0; 200.7)	**−6.0 (−7.3; − 4.8)**	193.5 (192.0; 195.0)				

Legend: CI-confidence interval; Q1: first quartile; Q2: second quartile; Q3: third quartile; Q4: fourth quartile; ^a^: categorisation at 75%-percentile; bold marked: significant differences

**Table 3:** Mean predictions of WD and TOH within the weather parameter categories and mean predicted differences between the categories stratified by men

Men Predicted mean (95% CI)	Q1	Difference Q2-Q1	Q2	Difference Q3-Q2	Q3	Difference Q4-Q3	Q4
**Temperature** (°C)	≤4.5		> 4.5- ≤ 12.9		> 12.8- ≤ 20.5		> 20.5
Daily WD (min)	90.2 (89.6; 90.8)	**5.7 (4.8; 6.6)**	95.9 (95.3; 96.5)	**5.9 (5.0; 6.8)**	101.9 (101.2; 102.5)	**6.1 (5.2; 6.9)**	108.1 (107.4; 108.7)
Daily TOH (min)	159.9 (158.2; 161.5)	**23.8 (21.5; 26.2)**	183.8 (182.1; 185.5)	**24.6 (22.2; 27.0)**	208.4 (206.7; 210.1)	**24.4 (22.1; 26.7)**	233.2 (231.4; 234.7)
**Radiation** (kWh/m^2^)	≤1170		> 1170- ≤ 2770		> 2770- ≤ 5190		> 5190
Daily WD (min)	92.3 (91.7; 92.9)	**3.0 (2.1; 3.8)**	95.3 (94.7; 96.0)	**5.0 (4.1; 5.8)**	100.1 (99.5; 100.8)	**8.3 (7.3; 9.2)**	108.4 (107.8; 109.1)
Daily TOH (min)	170.5 (168.8; 172.1)	**11.6 (9.3; 13.8)**	182.1 (180.4; 183.8)	**19.4 (17.1; 21.7)**	201.1 (199.4; 202.8)	**30.8 (28.3; 33.2)**	232.0 (230.3; 233.8)
**Sunshine** (h)	≤1.3		> 1.3- ≤ 5.0		> 5.0- ≤ 9.3		> 9.3
Daily WD (min)	92.0 (91.3; 92.6)	**4.2 (3.3; 5.0)**	96.2 (95.5; 96.8)	**4.7 (3.8; 5.6)**	100.8 (100.2; 101.5)	**6.2 (5.2; 7.1)**	107.0 (106.4; 107.6)
Daily TOH (min)	171.8 (170.2; 173.5)	**14.0 (11.7; 16.3)**	185.9 (184.2; 187.6)	**16.8 (14.5; 19.1)**	202.7 (201.0; 204.4)	**21.6 (19.2; 24.0)**	224.3 (222.6; 226.0)
**Humidity** (%)	≤80		> 80- ≤ 87		> 87- ≤ 94		> 94
Daily WD (min)	104.9 (104.3; 105.6)	**−4.5 (−5.3; −3.6)**	100.5 (99.8; 101.1)	**−2.9 (−3.8; −2.1)**	97.6 (97.0; 98.2)	**− 5.1 (− 5.9; − 4.2)**	92.6 (92.0; 93.2)
Daily TOH (min)	216.2 (214.6; 217.8)	**−15.3 (−17.6; −13.0)**	201.0 (199.3; 202.7)	**−10.2 (−12.4; −7.9)**	190.8 (189.2; 192.4)	**−15.7 (− 17.9; − 13.5)**	175.4 (173.7; 177.1)
**Windspeed** (m/s)	≤0.8		> 0.8- ≤ 1.3		> 1.3- ≤ 2.0		> 2.0
Daily WD (min)	101.5 (100.9; 102.1)	**−0.9 (−1.8; −0.1)**	100.5 (99.9; 101.1)	**−1.8 (−2.6; −0.9)**	98.8 (98.1; 99.4)	**− 4.1 (− 5.0; − 3.3)**	94.7 (94.1; 95.3)
Daily TOH (min)	209.0 (207.4; 210.5)	**−5.7 (−8.0; −3.4)**	203.0 (201.4; 204.7)	**− 8.3 (− 10.6; −6.0)**	194.8 (193.1; 196.5)	**−19.0 (− 21.3; − 16.7)**	175.9 (174.2; 177.6)
**Rain**^**a**^ (mm/h)	≤1.6		> 1.6				
Daily WD (min)	99.9 (99.5; 100.2)	**−3.7 (−4.2; − 3.2)**	96.2 (95.5; 96.8)				
Daily TOH (min)	197.8 (196.9; 198.8)	**−7.2 (−8.5; −5.8)**	190.7 (189.0; 192.3)				

Legend: CI-confidence interval; Q1: first quartile; Q2: second quartile; Q3: third quartile; Q4: fourth quartile; ^a^: categorisation at 75%-percentile; bold marked: significant differences

The highest increase in WD and TOH was recorded in the values of the solar radiation from Q3 (> 2770- ≤ 5190 kWh/m^2^) to Q4 (> 5190 kWh/m^2^) with an increase in WD of 8.3 min (95% CI: 7.3; 9.2) for men and 8.4 min (95% CI: 7.6; 9.3) for women. At this transition, men were on average out-of-home 30.8 min (95% CI: 28.3; 33.2) and women 23.6 min (95% CI: 21.4; 25.8) longer.

WD and TOH showed a continuous decrease for higher humidity, windspeed and precipitation values. The activity patterns for men and women were inconsistent for humidity. At the transition from the third highest (> 87- ≤ 94%) to the highest (> 94%) humidity values, men had the greatest decrease in WD of 5.1 min (95% CI: − 5.9; − 4.2) compared to 3.4 min (95% CI −4.3, − 2.4) for women for the same transition. Besides, there was the largest discrepancy between TOH decrease values in men and women at differences (Q2-Q1) and (Q4-Q3), where it was just over twice as high in men as in women (− 15.3 min [95% CI: − 17.6; − 13.0] vs. − 6.9 min [− 8.9; − 4.8] and − 15.7 min [− 17.9; − 13.5] vs. − 7.4 min [− 9.6; − 5.1], respectively).

The change to the fourth quartile (Q4-Q3) with windspeeds above 2 m/s showed the strongest decrease in WD in women (− 5.2 [95% CI: − 6.1; − 4.3]) as well as the strongest decrease in TOH in both sexes was recorded (men: − 19.0 min [95% CI: − 21.3; − 16.7], and women − 21.1 min [− 23.2; − 18.9]).

Similarly, on days with higher rainfall (> 1.6 mm/h), women walked 4.6 min (95% CI: − 5.1; − 4.1) and men 3.7 min (− 4.2; − 3.2) less. Outdoors, women spent 6.0 min (− 7.3; − 4.8) and men 7.2 min (− 8.5; − 5.8) less time.

**Fig. 2:**
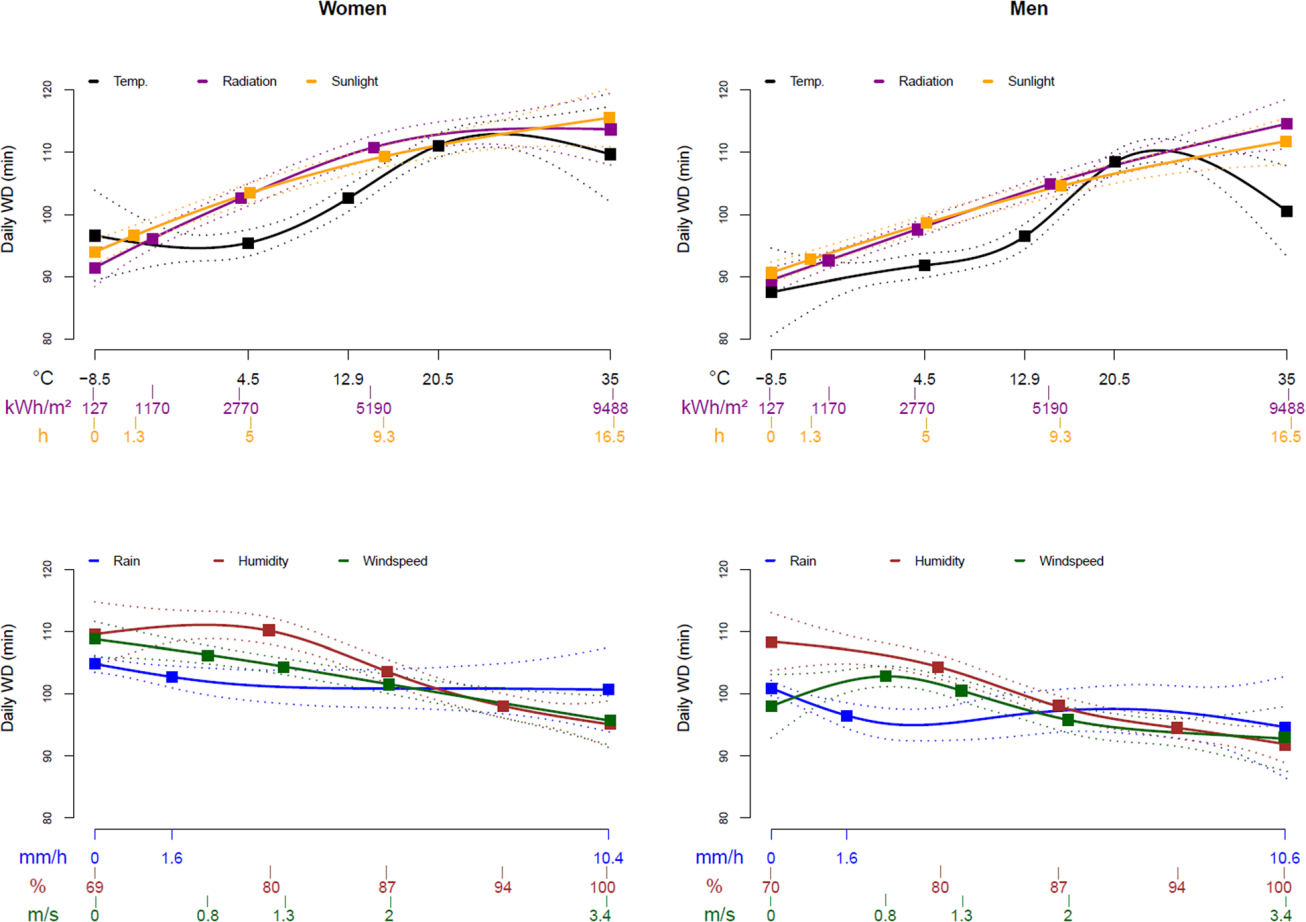
Association between different weather parameters and walking duration presented as cubic splines with 95%-pointwise confidence bands. Legend: Temp.-temperature; WD-walking duration; CB-pointwise confidence band; Exclusion of the upper 5% extreme values for rain and windspeed values and exclusion of the lower 5% extreme values for low humidity values; Colored boxes: percentile values at the category boundaries (exact values in Supplement Table A.3 and A.4)

**Fig. 3:**
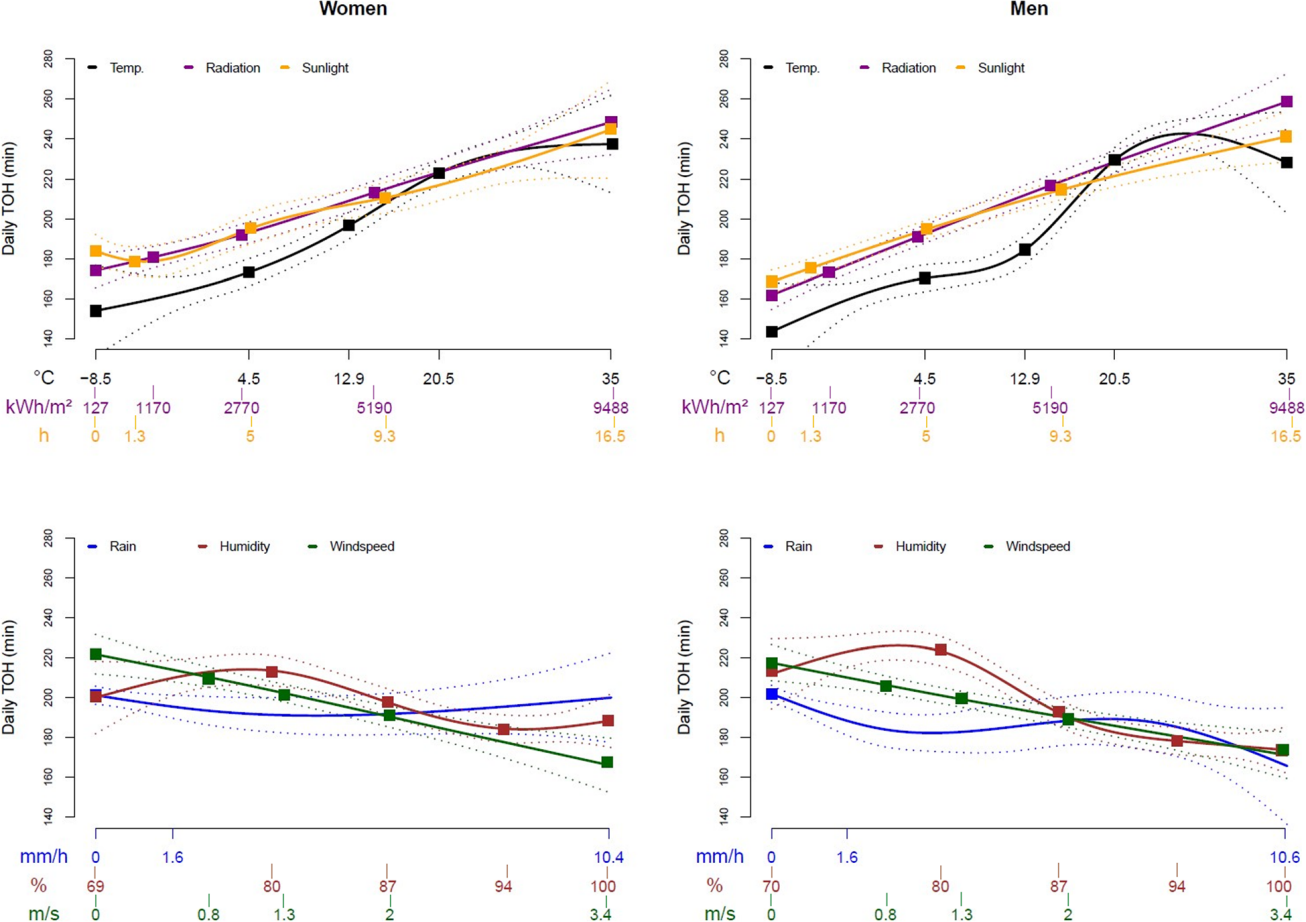
Association between different weather parameters and time out-of-home presented as cubic splines with 95%-pointwise confidence bands. Legend: Temp.-temperature; TOH-time out-of-home; CB-pointwise confidence band Exclusion of the upper 5% extreme values for rain and windspeed values and exclusion of the lower 5% extreme values for low humidity values; Colored boxes: percentile values at the category boundaries (exact values in Supplement Table A.3 and A.4)

In Fig. 2, the predictions for WD and in Fig. 3, the predictions for TOH from the continuous weather values are shown as cubic splines. In the top two graphs, the gender-stratified splines for temperature, solar radiation, and sunshine hours are plotted, whose WD and TOH predictions indicated a positive direction. For temperature, WD showed a peak at about 25 °C with a decrease afterwards, while in the two graphs below, the gender-stratified splines for humidity, windspeed, and rainfall are plotted, whose WD and TOH predictions indictated a more negative slope. Exact values related to the quartile bounderies are shown in Supplemental Tables A.3 and A.4.

## Discussion

In this large prospective cohort study with 1329 evaluable older adults from the ActiFE-study, we measured daily WD and daily TOH on up to 21 days for each participant at three observation periods during seven years of follow-up. In general, we observed that women walked significantly more than men per day. TOH on the other hand, was comparable in men and women. Incorporating weather data, we found that various weather parameters were strongly associated with participants’ WD and TOH. A particularly positive contribution to the increase in WD and TOH was seen for higher air temperature, higher solar radiation, and increased sunshine duration. There was a rather inverse contribution toward a decrease in WD and TOH, especially with increasing humidity, higher windspeeds and more rainfall.

Our results are in line with some previous studies that have also shown that higher temperatures and longer daytime significantly increase PA [8–10]. Similarly, Feinglass et al. using uniaxial accelerometers reported that light to heavy rainfall resulted in a reduction in PA [13]. Among 227 seniors from the Barcelona region, researchers found that regardless of the walkability of the neighbourhood, rain generally discourages them from walking more [16]. In our results, this was the case when we looked at the single WD predictions for the rainfall percentiles. Our spline curve also illustrated this, but took on a more constant shape in the region after the 75% percentile. Presumably, this might be due to the fact that there is often abundant precipitation of relatively short duration in the hot summer months, after which it is drier again. This could lead to a balancing effect between decreasing and increasing PA. However, for precipitation, TOH showed higher single predicted values on the highest precipitation days than on no precipitation days. Presumably, this behavior could possibly be explained by the fact that more time was spent in closed spaces outside home on rainy summer days. In a study from the United States with participants wearing accelerometers over a five-week period data showed less WD and increased sitting on colder or shorter days [17].

We noted from our results that the differences in predictions between weather quartiles at WD and TOH consistently had the same sign, and therefore showed the same trends when weather conditions changed. In addition, a study by Mikolaizak et al. and Rapp et al. of the same study population, but including only baseline data, also reached the similar conclusion that with more TOH, WD also increases, and vice versa. This finding is supported by other sensor-based studies [18–22].

Our study participants were largely retired or transitioned to retirement during the follow-up. Retirement might have altered physical activity compared to the working years. In a study of nearly 5800 participants from the United States, those who transitioned to full-time retirement were among the least active, compared with those who were still working, transitioned to part-time work or entered unemployment [23]. A partially opposite conclusion was reached in a French study, which found that retirement was associated with both an increase in physical activity as well as an increase in time spent in front of the television [24].

On the one hand, older people might leave their home less often and might spend more time indoors when they are away from home, affecting their daily proportion of TOH. Younger people, instead, may be more independent of the weather because their work requires them to go outside during the week, while older people are more dependent on external conditions to go outside. Therefore, TOH might increase after retirement as they are no longer obligated to a job. There is also evidence that functional status is associated with WD [25]. Interestingly, WD increased with functional status only until a certain threshold. It seems that for frail older people, mainly their frailty status affects their activity levels, while for fit healthy older people, other factors are more important, including environmental factors such as weather conditions. However, there are also weather conditions which have a stronger effect on people with certain health conditions, such as higher temperature and humidity on people with cardiac and respiratory diseases [26, 27]. The evaluation of environmental, social, physical or psychological factors as mediators of the observed association between weather conditions and level of physical activity in older adults is a research question of interest, which should be addressed in the future.

The study has several strengths. We were able to include a high number of participants in the analyses, even up to the second follow-up, allowing us to record activity changes over up to seven years. This resulted in a very high number of observed days, for which we also had almost complete weather data available.

A limitation of this study is that it is not exactly clear how long each participant walked during their TOH. Although it was asked which different activities were performed during this time, no estimated time of walking was given. It is possible that there is some sort of bias in the times, as it may have occurred that participants were animated to move and go out more than usual due to study participation. In addition, our study population is limited to one region in Germany. Weather data for each subject and day came from the same weather station. Depending on the distance from the exact place of residence and the locations away from home, inaccuracies in the weather values may have occurred here as well. Presumably, our results are only applicable to older people in the middle European temperature latitudes, as WD and TOH behaviour under different weather conditions may vary among different cultures.

For better generalizability, we set TOH to zero on days when participants were not outside. Similarly, we also set TOH to zero on days when the information on TOH in the movement diary was not recorded at baseline, as done in the work of Rapp et al. [19] There might also well have been misclassifications with regard to these missing days. Thus, there were 2862 days (18.6%) out of 15,392 where participants spent time only within their homes.

In future analysis, it might be interesting to analyse the association between PA and TOH in interaction with weather parameters. Here, we might explore the question of whether weather has an effect on the time walked during TOH.

## Conclusion

In conclusion, our results support and extend the available literature suggesting that long, sunny, and warm days were associated with participants’ behaviour to be out of the house more frequently and to walk for a longer time. Days that were very rainy, humid and windy had the opposite associations. Therefore, it would be highly advisable for future analyses related to outdoor movement data to include specific weather parameters and possibly adjust for them.

## Supplementary Information

**Additional file 1: Table A.1.** Association between the weather parameter values calculated by Spearman’s rank correlation coefficients. **Table A.2.** Regression coefficients (95% confidence interval (CI)) and explained variance (adjusted for sex and age) for mutually adjusted model of WD and TOH with weather parameters. **Table A.3.** Prediction of walking duration and time out-of-home for weather quartile boundary values for women. **Table A.4.** Prediction of walking duration and time out-of-home for weather quartile boundary values for men

## Data Availability

The data that support the findings of this study are available from the ActiFE-Ulm study group but restrictions apply to the availability of these data, which were used under license for the current study, and so are not publicly available. Data are however available from the authors upon reasonable request and with permission from the ActiFE-Ulm study group.

## Abbreviations

WD: Walking duration
PA: Physical activity
TOH: Time out-of-home
ActiFE: Acitvity and Function in Older People in Ulm
GMM: Generalized mixed model
GAM: Generalized additive model
