# Supplementary material for: The relationship of weather with daily physical activity and the time spent out of home in older adults from Germany – the ActiFE study
Source: Eur Rev Aging Phys Act. 2022 Feb 12;19:6. doi: 10.1186/s11556-022-00286-0 (PMC8903592; doi:10.1186/s11556-022-00286-0)
Supplement: Supplementary file 1 — Additional file 1: Table A.1. Association between the weather parameter values calculated by Spearman’s rank correlation coefficients. Table A.2. Regression coefficients (95% confidence interval (CI)) and explained variance (adjusted for sex and age) for mutually adjusted model of WD and TOH with weather parameters. Table A.3. Prediction of walking duration and time out-of-home for weather quartile boundary values for women. Table A.4. Prediction of walking duration and time out-of-home for weather quartile boundary values for men [file 11556_2022_286_MOESM1_ESM.docx]

**SUPPLEMENTARY MATERIAL**

**Table A.1: Association between the weather parameter values calculated by Spearman´s rank correlation coefficients.**

| Spearman´s rank correlation coefficient | Temperature | Solar radiation | Sunshine duration | Humidity | Windspeed | Rain |
| --- | --- | --- | --- | --- | --- | --- |
| Temperature | - | **0.79** | **0.64** | -0.49 | -0.39 | -0.02 |
| Solar radiation |  | - | **0.91** | **-0.78** | -0.25 | -0.26 |
| Sunshine duration |  |  | - | **-0.78** | -0.24 | -0.40 |
| Humidity |  |  |  | - | -0.06 | 0.36 |
| Windspeed |  |  |  |  | - | 0.24 |
| Rain |  |  |  |  |  | - |

Legend: All significant with p<0.05; bold marked: high correlations with rho >0.50 and rho <0.50

**Table A.2: Regression coefficients (95% confidence interval (CI)) and explained variance (adjusted for sex and age) for mutually adjusted model of WD and TOH with weather parameters.**

| **Walking duration** | Adjusted R^2^ | Temperature | Rain | Windspeed |
| --- | --- | --- | --- | --- |
| Basic models (single weather parameter) | 0.104 | 0.73 (0.66; 0.81) | - | - |
|  | 0.086 | - | -0.61 (-0.78; -0.44) | - |
|  | 0.088 | - | - | -3.46 (-4.24; -2.68) |
| Model 1a (Temperature and rain) | 0.108 | 0.76 (0.68; 0.83) | -0.73 (-0.89; -0.56) | - |
| Model 1b (Temperature and windspeed) | 0.104 | 0.71 (0.62; 0.79) | - | -0.70 (-1.54; 0.13) |
| Model 2 (all weather parameters) | 0.108 | 0.74 (0.66; 0.83) | -0.72 (-0.89; -0.55) | -0.32 (-1.16; 0.52) |
| **Time out-of home** | | | | |
| Basic models (single weather parameter) | 0.074 | 2.87 (2.60; 3.13) | - | - |
|  | 0.046 | - | -1.02 (-1.61; -0.43) | - |
|  | 0.053 | - | - | -15.36 (-18.05; -12.67) |
| Model 1a (temperature and rain) | 0.075 | 2.91 (2.64; 3.18) | -1.48 (-2.06; -0.89) | - |
| Model 1b (temperature and windspeed) | 0.074 | 2.67 (2.38; 2.96) | - | -5.04 (-7.93; -2.15) |
| Model 2 (all weather parameters) | 0.076 | 2.74 (2.45; 3.03) | -1.38 (-1.97; -0.79) | -4.30 (-7.20; -1.39) |

**Table A.3: Prediction of walking duration and time out-of-home for weather quartile boundary values for women.**

| **Women**  Prediction (95% CI) | Min | Q1 | Q2 | Q3 | Max |
| --- | --- | --- | --- | --- | --- |
| **Temperature** (°C)  Daily WD (min)  Daily TOH (min) | -8.5 °C  96.6 (89.2; 104.0)  153.9 (130.7; 177.1) | 4.5 °C  95.4 (93.3; 97.6)  173.3 (166.3; 180.2) | 12.9 °C  102.6 (100.5; 104.8)  196.6 (189.8; 203.4) | 20.5 °C  111.1 (109.2; 113.1)  223.0 (216.6; 229.4) | 35.0 °C  109.7 (101.8; 117.5)  237.4 (212.4; 262.4) |
| **Radiation** (kWh/m²)  Daily WD (min)  Daily TOH (min) | 127 kW/m²  91.4 (88.4; 94.5)  174.1 (165.3; 182.8) | 1170 kWh/m²  96.1 (94.5; 97.8)  180.7 (175.2; 186.2) | 2770 kWh/m²  102.6 (100.8; 104.4)  192.0 (186.7; 197.3) | 5190 kWh/²  110.8 (108.9; 112.8)  213.2 (207.2; 219.2) | 9488 kWh/m²  113.7 (107.9; 119.6)  248.5 (231.8; 265.2) |
| **Sunlight** (h)  Daily WD (min)  Daily TOH (min) | 0.0 h  94.0 (91.9; 96.1)  183.7 (175.1; 192.2) | 1.3 h  96.7 (95.1; 98.3)  178.7 (171.2; 186.2) | 5.0 h  103.5 (101.9; 105.2)  195.3 (187.4; 203.2) | 9.3 h  109.4 (107.7; 111.2)  210.6 (202.7; 218.6) | 16.5 h  115.6 (110.8; 120.4)  244.9 (219.8; 270.0) |
| **Humidity** (%)  Daily WD (min)  Daily TOH (min) | 69 %  109.6 (104.5; 114.8)  200.4 (182.3; 218.4) | 80 %  109.8 (107.6; 111.9)  213.0 (205.4; 220.6) | 87 %  103.1 (101.1; 105.1)  196.2 (189.2; 203.1) | 94 %  97.8 (95.7; 99.8)  184.2 (177.1; 191.2) | 100 %  95.1 (91.1; 99.0)  188.4 (174.6; 202.2) |
| **Windspeed** (m/s)  Daily WD (min)  Daily TOH (min) | 0.0 m/s  108.8 (105.9; 111.7)  221.7 (211.6; 231.7) | 0.8 m/s  106.1 (104.6; 107.5)  209.5 (204.5; 214.4) | 1.3 m/s  104.2 (102.9; 105.5)  201.4 (196.8; 205.9) | 2.0 m/s  101.3 (99.7; 102.9)  189.5 (184.0; 195.0) | 3.4 m/s  95.7 (91.7; 99.7)  166.2 (152.3; 180.0) |
| **Rain^a^** (mm/h)  Daily WD (min)  Daily TOH (min) | 0.0 mm/h  104.8 (103.4; 106.2)  201.2 (196.5; 205.8) | p75: 1.6 mm/h  102.7 (100.8; 104.5)  200.0 (177.0; 222.9) | Max: 10.4 mm/h  100.7 (93.6; 107.7)  223.0 (216.6; 229.4) |  | |

Legend: CI - confidence interval; Min: Minimum; Q1: first quartile; Q2: second quartile; Q3: third quartile; Max: Maxiumum; **^a^**: categorisation at 75% percentile; p: percentile

**Table A.4: Prediction of walking duration and time out-of-home for weather quartile boundary values for men.**

| **Men**  Prediction (95% CI) | Min | Q1 | Q2 | Q3 | Max |
| --- | --- | --- | --- | --- | --- |
| **Temperature** (°C)  Daily WD (min)  Daily TOH (min) | -8.5 °C  87.5 (80.3; 94.7)  143.5 (118.5; 168.6) | 4.5 °C  91.8 (89.9; 93.8)  170.3 (163.4; 177.2) | 12.9 °C  96.5 (94.4; 98.6)  184.5 (177.2; 191.8) | 20.5 °C  108.5 (106.6; 110.3)  229.7 (223.3; 236.2) | 35.0 °C  100.5 (93.1; 108.0)  228.1 (202.1; 254.1) |
| **Radiation** (kWh/m²)  Daily WD (min)  Daily TOH (min) | 127 kW/m²  89.4 (87.3; 91.6)  161.8 (154.5; 169.2) | 1170 kWh/m²  92.6 (91.3; 94.0)  173.2 (168.5; 178.0) | 2770 kWh/m²  97.6 (96.3; 98.9)  190.8 (186.2; 195.4) | 5190 kWh/²  104.9 (103.4; 106.4)  216.7 (211.4; 222.0) | 9488 kWh/m²  114.6 (110.5; 118.7)  258.7 (244.5; 273.0) |
| **Sunlight** (h)  Daily WD (min)  Daily TOH (min) | 0.0 h  90.6 (88.9; 92.4)  168.5 (162.4; 174.5) | 1.3 h  92.8 (91.4; 94.2)  175.5 (170.7; 180.4) | 5.0 h  98.7 (97.3; 100.0)  194.9 (190.2; 199.5) | 9.3 h  104.7 (103.2; 106.1)  214.7 (209.6; 219.8) | 16.5 h  111.8 (108.0; 115.7)  241.3 (228.3; 254.3) |
| **Humidity** (%)  Daily WD (min)  Daily TOH (min) | 70 %  108.3 (103.8; 112.7)  213.2 (196.6; 229.8) | 80 %  103.9 (101.9; 105.8)  221.9 (214.6; 229.2) | 87 %  97.6 (95.8; 99.3)  190.3 (183.4; 197.1) | 94 %  94.3 (92.5; 96.1)  177.8 (170.9; 184.8) | 100 %  91.8 (88.7; 95.0)  173.6 (161.7; 185.6) |
| **Windspeed** (m/s)  Daily WD (min)  Daily TOH (min) | 0.0 m/s  98.0 (92.8; 103.2)  217.4 (208.2; 226.6) | 0.8 m/s  102.8 (101.1; 104.5)  205.6 (201.1; 210.0) | 1.3 m/s  100.1 (98.3; 101.9)  198.4 (194.3; 202.5) | 2.0 m/s  95.6 (93.4; 97.8)  189.1 (184.2; 194.1) | 3.4 m/s  93.6 (90.9; 96.2)  178.7 (170.9; 186.5) |
| **Rain^a^** (mm/h)  Daily WD (min)  Daily TOH (min) | 0.0 mm/h  100.9 (99.7; 102.2)  201.8 (197.4; 206.2) | p75: 1.6 mm/h  96.4 (94.3; 98.5)  165.7 (135.7; 195.6) | Max: 10.6 mm/h  94.6 (86.1; 103.0)  229.7 (223.3; 236.2) |  | |

Legend: CI - confidence interval; Min: Minimum; Q1: first quartile; Q2: second quartile; Q3: third quartile; Max: Maxiumum; **^a^**: categorisation at 75% percentile; p: percentile
